# Chronic recurrent multifocal osteomyelitis: Case report and review of the literature

**DOI:** 10.1097/MD.0000000000038850

**Published:** 2024-07-26

**Authors:** Lin Liu, Ranran Zhang, Nana Nie, Dahai Wang, Yi Lin, Zhaisong Gao, Hong Chang

**Affiliations:** a Department of Pediatric Nephrology, Rheumatology, and Immunology, The Affiliated Hospital of Qingdao University, Qingdao, China.

**Keywords:** bone biopsy, children, chronic recurrent multifocal osteomyelitis, diagnosis, magnetic resonance imaging

## Abstract

**Backgrounds:**

Chronic recurrent multifocal osteomyelitis (CRMO) is a rare inflammatory disease.

**Objective:**

This report aims to analyze the clinical characteristics of CRMO and enhance clinicians’ comprehension. We present 3 atypical cases, highlighting their unique clinical features, diagnostic challenges, and effective treatment strategies.

**Methods:**

We retrieved 3 CRMO cases in our hospital from September 2019 to August 2022. The clinical features were analyzed retrospectively, and relevant literatures were reviewed.

**Results:**

All 3 cases initially presented with bone pain, normal leucocyte counts, negative rheumatoid factors and no signs of sclerotic or hyperostotic lesions. Case 1, a 12-year-old girl, exhibited concurrent acne on the forehead and historic necrotizing lymphadenitis, a previously unreported association with CRMO. Case 2, a 14-year-old boy, tested positive for human leukocyte antigen-B27 and displayed scoliosis along with multifocal osteomyelitis. Case 3, a 9-year-old girl, presented with scoliosis, and chest computed tomography revealed changes in the T8 vertebral body, initially suggesting Langerhans cell histiocytosis. Bone biopsy was conducted in case 1 and case 3, revealing chronic inflammation. All 3 cases affected long bones, pelvis, and vertebra, involving 8, 6 and 5 bones, respectively, identified by magnetic resonance imaging. Genetic analysis was undertaken in cases 1 and 2 but no pathogenic mutations were identified. Upon the confirmation of a CRMO diagnosis, all patients were initiated on a treatment regimen comprising nonsteroidal anti-inflammatory drugs and tumor necrosis factor-α inhibitors. In cases 1 and 2, due to the severity of their bone pain, they were also administered to disease-modifying anti-rheumatic drugs, specifically methotrexate. All 3 patients achieved remission of bone pain. To gain a more comprehensive understanding of CRMO, we conducted a thorough review of relevant literature.

**Conclusion:**

CRMO is a rare autoinflammatory bone disorder with diverse clinical presentations and a lack of specific laboratory tests, which leads to potency to misdiagnosis or delayed diagnosis. By raising awareness and improving diagnostic criteria, physicians are now better equipped to identify CRMO. We contribute to share our understanding of CRMO by presenting 3 cases with untypical clinical features, highlighting the importance of recognizing this rare condition for timely and effective management.

## 1. Introduction

Chronic recurrent multifocal osteomyelitis (CRMO), also known as chronic nonbacterial osteomyelitis (CNO), is a rare inflammatory bone disease primarily affecting children and adolescents.^[1]^ It was estimated to occur at a rate of approximately 4/1,000,000, commonly manifests in individuals between the ages of 10 and 12 but can affect individuals of all age groups.^[2,3]^ It predominantly affect females, with a male-to-female ratio of approximately 1:4 to 1:1.5.^[4]^ CRMO is characterized by recurrent episodes of bone pain, mainly affecting the lower extremities, often accompanied by symptoms like fever, fatigue, and weight loss.^[5–7]^ In some cases, it can also involve other organs such as the skin and intestines.^[7,8]^ Due to the lack of typical symptoms or definitive laboratory tests, CRMO is often misdiagnosed or diagnosed with a delay. The diagnosis typically relies upon the exclusion of infection, malignancy and other potential underlying causes. Currently, there are no established consensus guidelines for CRMO treatment, and the clinical approaches are largely based on empirical evidence. Presently, clinical doctors are lack sufficient knowledgeable about CRMO, frequently resulting in misdiagnosis or delayed diagnosis, which can have adverse implications. Therefore, this article aims to analyze the clinical manifestations, diagnosis, and treatment process of 3 CRMO patients diagnosed at our hospital. By doing so, we seek to enhance clinicians’ understanding of the disease and expedite patients’ access to suitable diagnosis and treatment.

## 2. Case presentations

### 2.1. Case 1

A 12-year-old girl presented with a one-month history of intermittent fever and bone pain. She experienced fever episodes, without any apparent triggers, that lasted for 3 to 5 days each time, peaking in the afternoon. The bone pain, which initially affected her spine, gradually spread to her waist, right hip, and right knee. Although no redness or swelling was observed, she complained of significant weakness in her extremities, particularly her lower limbs. No special treatment was given before she admitted to our hospital on September 5, 2019.

Her medical history included a diagnosis of epilepsy 4 years prior, which had been well-controlled with oxcarbazepine and levetiracetam for 2 years. She had discontinued medication for the past 2 years without any issues. Her family and personal history were unremarkable.

**Figure 1.**
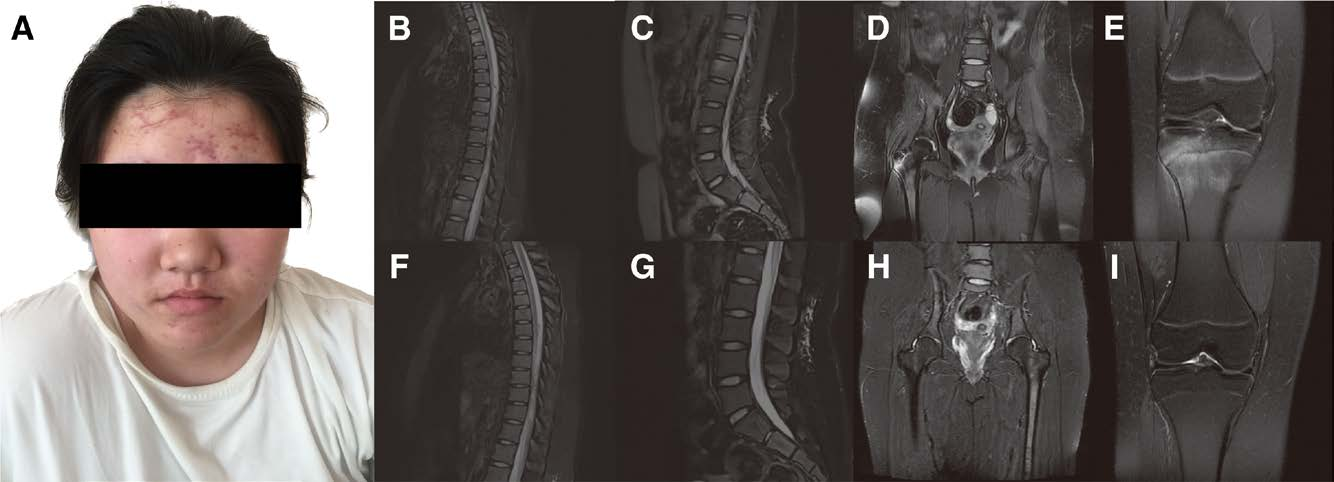
(a) Facial acne on the forehead. (b) Before treatment, the MR scan of the thoracic vertebra showed high signals in the T2 lipid sequence. (c) Before treatment, the MR scan of the lumbar vertebral showed high signals in the lipid sequence. (d) Before treatment, the MR scan of the hip joint showed high signals between left femoral trochanters. (e) Before treatment, the MR scan of the right knee joint showed multiple patchy and strip high signals in the bone marrow cavities of femur and tibia. (b) Thoracic vertebral, (c) lumbar vertebral, (d) hip joint, (e) right knee joint. (f–i) After treatment, the original abnormal imaging signal disappeared. (f) Thoracic vertebral, (g) lumbar vertebral, (h) hip joint, (i) right knee joint.

Upon examination, her face was found to be covered with densely distributed red papules, pustules, nodules, and scars (Fig. 1a). Her cardiac, pulmonary, abdominal, and neurological examinations were normal. Her muscular strength and tone in both upper and lower extremities are within the normal range. However, a notable tenderness is present in her left knee joint, despite the absence of any redness, swelling, or increased skin temperature.

The blood tests revealed elevated inflammatory markers (C-reaction protein [CRP]: 37.08 mg/L, erythrocyte sedimentation rate (ESR): 97 mm/h). Magnetic resonance imaging (MRI) showed high signals on the T2 lipid sequence in the thoracic and lumbar vertebrae, as well as in the bone marrow cavities of both femur and tibia (Figs. 1b–e). To exclude metastatic tumors, chest, upper and lower abdominal computed tomography (CT) scans, and urinary system ultrasound examinations were performed, revealing no apparent abnormalities. Bone marrow puncture showed an increased plasma cell proportion with negative culture results. Despite anti-infective therapy with meropenem and vancomycin, the patient continued to experience recurrent fever and bone pain. Positron emission tomography/computed tomography imaging showed lymph node enlargement in the left neck and increased metabolism in multiple places in the skeleton. Cervical lymph node biopsy indicated necrotizing lymphoid lesions, strongly suggesting historical necrotizing lymphadenitis. A definitive diagnosis required a bone biopsy, which showed chronic inflammation and ruled out Langerhans cell histiocytosis (LCH), suppurative infection, or malignancy, strongly supporting a diagnosis of CRMO. Additionally, whole exome sequencing detected FA2H and RFWD3 gene deletions; however, no known pathogenic association with the disease was identified.

Taking into account all the information, the patient was ultimately diagnosed as CRMO, and treated with tumor necrosis factor (TNF)-α inhibitors, methotrexate, corticosteroid, and nonsteroidal anti-inflammatory drugs (NSAIDs) sequentially. Improvement of symptoms was achieved gradually, including alleviation of bone pain, normalization of body temperature, and a significant reduction in facial acne. Correspondingly, the inflammatory markers including ESR and CRP decreased to normal, and the original abnormal imaging signals disappeared (Figs. 1f–i). The patient continues to visit the outpatient department for regular follow-up appointments, adjusted the dosage and frequency of drug administration based on laboratory and imaging test results. Currently, she is receiving maintenance treatment with a subcutaneous injection of 40 mg adalimumab biweekly and oral 10 mg methotrexate weekly.

### 2.2. Case 2

A 14-year-old boy presented with a two-month history of bone pain, primarily located in his lower left limb, without fever. Initially, the pain affected his left knee and left hip, causing limping, especially in the morning. However, over time, the pain progressively extended to both ankles and the right knee. The child’s hip, knee, and ankle joints displayed no indications of redness, swelling, or an increase in skin temperature. Nevertheless, he presented with limb weakness, predominantly affecting the lower extremities. After seeking treatment at a local hospital, where he was prescribed a seven-day course of oral ibuprofen, he initially experienced some relief. However, his symptoms gradually worsened over time. He was admitted to our hospital on April 27, 2020. The patient had no significant personal or family medical history.

During the physical examination, tenderness was observed in the sacroiliac and knee joints, as well as both plantar areas. No signs of redness or swelling were present and his muscle strength and tone in all 4 limbs appear normal. The Patrick sign was positive. Cardiac, pulmonary, abdominal, and neurologic examinations were unremarkable.

**Figure 2.**
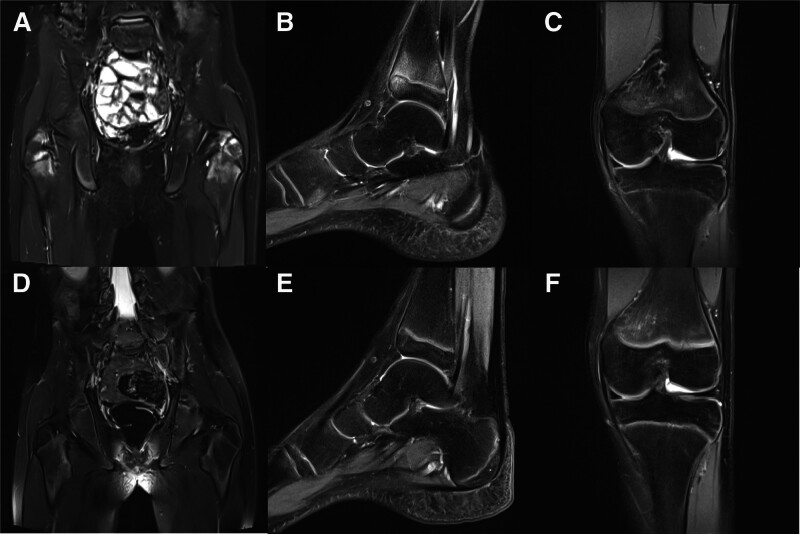
(a) Before treatment, the MR scan of the hip joint showed patchy high signals in the bilateral acetabulum and upper femur metaphysis in the T2 lipid sequence. (b) Before treatment, the MR scan of the left ankle joint showed high signals in the left tibial metaphysis and bone marrow cavity in the T2 lipid sequence. (c) Before treatment, the MR scan of the left knee joint showed patchy high signals in the bone marrow cavities of femur, and tibia. (d–f) After treatment, the original abnormal imaging signals were reduced. (d) Hip joint, (e) left ankle, (f) left knee joint.

The laboratory tests revealed mildly elevated inflammatory markers (CRP: 9.44 mg/L, ESR: 26 mm/h) and a positive result for human leukocyte antigen-B27 at 99.5%. To assess the overall skeletal condition, whole-body imaging was conducted, revealing localized uptake of imaging agents in the upper tibia on both sides and scoliosis. The agglomerative changes observed in the whole-body imaging are consistent with age-related findings. MR scan showed patchy high signals in the bilateral acetabulum, femur and tibia (Fig. 2). Whole exome sequencing did not identify any pathogenic mutations related to the disease.

The boy received a combination of NSAIDs, immunosuppressive drugs, and disease-modifying anti-rheumatic drugs (DMARDs), resulting in significant pain relief and improved mobility. However, following discharge, he experienced a relapse with elevated inflammatory markers. Additional treatment with corticosteroids and TNF-α inhibitors further improved the condition, and imaging revealed a reduction in lesion size. Currently, the patient is receiving ongoing treatment with subcutaneous injection of 40 mg adalimumab biweekly, oral 15 mg methotrexate every 2 weeks and 50 mg diclofenac sodium enteric-coated tablets twice a day.

### 2.3. Case 3

A 9-year-old girl presented with a two-month history of left costal pain, accompanied by shoulder height inequality but no signs of redness or swelling. She had initially sought treatment at another hospital, where chest CT showed changes in the T8 vertebral body, suggesting Langerhans cell histiocytosis. X-ray revealed scoliosis and morphological changes in the T8 vertebral body. Subsequently, she was admitted to our hospital due to suspected spinal tumor (T8 space occupation), pathological thoracic fracture, and scoliosis. Initial blood tests, including routine, CRP, ESR, coagulation, liver, and kidney function, anti-streptolysin O, and rheumatoid factor, did not reveal any abnormalities.

The patient was admitted to our hospital on August 24, 2022 for further treatment. And she was previously in good health and had no special personal or family history.

Upon examination, a 10 cm scar was visible on the patient’s back, accompanied by shoulder height inequality. The spine exhibits palpable scoliosis, yet there is no significant tenderness upon palpation. There were no limb malformations, and muscle strength and tone in the limbs was normal, with a negative Patrick sign. Cardiac, pulmonary, and abdominal examinations revealed no positive signs.

Relevant auxiliary tests were conducted, revealing an S-shaped curve with unnatural curvature in the anterior and lateral views of the entire spine, along with compensatory changes observed in various intervertebral spaces and vertebral bodies.

On June 30, 2022, under general anesthesia, an incision biopsy of the spinal bone tumor and muscle suture was performed. Examination of the thoracic vertebra tissue revealed chronic inflammation, with immunohistochemical results showing Langerin (‐), CD1a (‐), CD68 (+ in small amounts), CD163 (+ in small amounts), and S100 (‐). Following discharge, the patient experienced left rib pain that initially responded to NSAID medication but later worsened. Enhanced thoracic MRI indicated T8 hypersignal with prevertebral inflammatory changes, while ECT suggested arthritis in the T8/T9 vertebral body and the right sacroiliac joint. MRI of the sacroiliac and knee joints showed sacroiliac arthritis, knee arthritis, and right sacral bone marrow edema.

Based on clinical manifestations and auxiliary examinations, the patient received a diagnosis of CRMO and was treated with NSAIDs and adalimumab. Subsequent tests revealed abnormal curvature in the spine and compensatory changes in several intervertebral spaces and vertebral bodies. As a result, the patient received 3 intravenous injections of 2.4 mg zoledronate sodium. Currently, the patient continues to receive maintenance treatment with a subcutaneous injection of 40 mg adalimumab biweekly without experiencing bone pain, and inflammatory indicators remain within normal limits.

## 3. Discussion

CRMO, initially described in 1972 by Giedion, is characterized by multifocal bone lesions with subacute and chronic symmetrical osteomyelitis, mainly affecting the long bones.^[9,10]^ Until now, only around 2000 cases all over the world were reported in the literature, with a higher incidence in Western countries, particularly in Europe.^[6,7]^ The limited recognition of CRMO and the absence of standardized diagnostic criteria likely result in underreporting and underestimation of its prevalence.^[2,11]^

Osteolytic changes may occur initially, with hyperplastic changes developing decades after the onset, and sclerotic or hyperostotic lesions may coexist in advanced patients.^[1,12]^ Multifocal bone pain is the hallmark symptom of CRMO, with a wide range of symptoms from mild, unspecific bone pain to localized swelling and warmth, and even severe pain.^[1,5,6]^ The clinical course is unpredictable, characterized by acute exacerbations and spontaneous remissions. Lesions can develop in any bone, with common sites including the metaphysis of long bones, the pelvis, spine, clavicles, and mandible. Cranial involvement is relatively rare.^[13–15]^ In the aforementioned cases, bone pain was the primary clinical symptom, primarily affecting the metaphysis of long bones, the pelvis, and vertebrae (Table 1). In case 3 bone destruction and bone marrow edema coexisted. The nonbacterial osteitis scores of 3 cases were 36, 39, and 34 points, respectively.^[12]^ Unlike some cases described in the literature, our patients did not exhibit sclerotic or hyperostotic lesions, possibly due to the relatively short disease duration and timely diagnosis (2–3 months from onset to diagnosis). Symmetrical involvement of bony lesions is seen in about half of the patients reported in the literature, and the bone lesions of our 3 patients also exhibited symmetrical accumulation.

**Table 1:** The clinical characteristics, therapy and prognosis of 3 children with CRMO.

	Case 1	Case 2	Case 3
Sex/age at disease onset (years)	12F	14M	9F
Duration before diagnosis (months)	2	2	3
Fever	+	-	-
Bone involved	Long bone femur, tibia Spine thoracic vertebra, lumbar vertebra Pelvis sacrum, ilium, pubis, ischium	Long bone femur, tibia Spine thoracic vertebra, lumbar vertebra Pelvis sacrum, ilium	Long bone femur, tibia Spine thoracic vertebra Pelvis sacrum, ilium
Complication	Necrotizing lymphadenitis	Scoliosis	Pathological thoracic fracture, scoliosis
Laboratory test			
White blood cell count	6.16 × 10^9^/L	7.39 × 10^9^/L	6.05 × 10^9^/L
C-reactive protein (mg/L)	37.08	9.44	2.26
Erythrocyte sedimentation rate (mm/h)	97.00	26.00	10.00
Rheumatoid factor	-	-	-
Cell factor	No data	-	No data
Bone marrow biopsy	-	No data	-
Pathogen	-	-	-
Human Leukocyte Antigen-B27	-	+	No data
Imaging examination			
X ray	No data	No data	T8 vertebral body morphological changes, scoliosis
Magnetic resonance imaging	High signals in the lipid sequence in the thoracic and lumbar vertebra High signals between left femoral trochanters Multiple patchy and strip high signals in the bone marrow cavities of femur and tibia	High signals in the bilateral acetabulum in the T2 lipid sequence High signals in the metaphysis and bone marrow cavity of femur, and tibia	T8 hypersignal with prevertebral inflammatory changes
Bone biopsy	Fibrosis and chronic inflammation	No data	Chronic inflammatory cell infiltration
Genetic test	No pathogenic mutations	No pathogenic mutations	No data
Initial suspicion	Infectious osteomyelitis	Juvenile idiopathic arthritis	LCH
Nonbacterial Osteitis scores	36	39	34
Therapy			
Nonsteroidal anti-inflammatory drugs	Ibuprofen, diclofenac sodium	Ibuprofen, diclofenac sodium	Etoricoxib, celecoxib
Disease-modifying anti-rheumatic drugs	Methotrexate	Methotrexate	-
Corticosteroids	+	+	-
Biological agents	Tumor necrosis factor-α inhibitors, adalimumab	Adalimumab	Adalimumab
Bisphosphate	-	-	+
Prognosis			
Follow-up (months)	46	39	15
Recurrence frequency	0	1, due to self-disuse adalimumab	0

Additionally, some extra-articular manifestations associated with CRMO have been described, including skin and gastrointestinal involvement, manifesting as inflammatory bowel disease, acne, psoriasis, and palmoplantar pustulosis.^[4,16]^ Acne and palmoplantar pustulosis frequently occur alongside synovitis, hyperostosis, and osteitis, collectively referred to as SAPHO syndrome. This has led to speculation that CRMO in children corresponds to SAPHO syndrome in adults, suggesting a potential clinical spectrum.^[4,7,17]^ Case 1 who exhibited skin involvement in the form of facial acne. Following a diagnosis of CRMO and subsequent treatment, her skin condition has undergone remarkable improvement. It is worth noting that, she was complicated with necrotizing lymphadenitis, an immune disease that can be accompanied by abnormal immune factors. And there is no literature report on CRMO combined with necrotizing lymphadenitis, both domestically and internationally. Since CRMO is an autoinflammatory disease, we speculated that necrotizing lymphadenitis can be accompanied by CRMO. Whether there is a correlation between them requires to be further studied.

The exact etiology of CRMO remains incompletely understood. It has been suggested that the imbalance between pro-inflammatory cytokines and anti-inflammatory cytokines may contribute to the abnormal activity of osteoclasts.^[7,18]^ Recent findings demonstrate that genetic, environmental, and immune factors may interact to lead to disease development. Elevated levels of pro-inflammatory cytokines, including IL-1, IL-6, TNF-α, and IL-20, have been identified in mononuclear macrophages, while anti-inflammatory cytokines like IL-10 and IL-19 exhibit lower expression.^[4,5]^ These findings have led to the up-regulation of the Nod-like receptor family pyrin domain containing 3 inflammasome transcript levels, resulting in an enhanced expression of IL-1β. This inflammatory cascade is believed to play a role in osteoclast activation through interactions between the receptor activator of nuclear factor-κB and its soluble ligand RANKL on osteoclast precursor cells, providing a theoretical basis for TNF targeting therapy.^[19–21]^

Laboratory findings in CRMO are generally nonspecific, with mild elevations in ESR and CRP levels.^[15]^ These markers do not predict disease course or severity.^[21]^ In our analysis of case 1 and case 2, we observed a elevation in the levels of ESR and CRP, both initially and during the active stages of the disease. This observation suggests that ESR and CRP maybe serve as cues of disease activity. Imaging plays a crucial role in diagnosis and detection.^[22]^ X-ray may remain normal in early stages but can eventually reveal nonspecific osteolytic or sclerotic changes.^[16]^ MRI is highly sensitive in identifying of bone lesions and tissue edema in early stages, even before erosions or sclerosis become apparent, characterized by increased signal on T2 weighted images and reduced signal on T1 weighted images. It is commonly used for assessing disease activity during follow-up and identifying and monitoring disease-related sequelae without radiation exposure.^[8,14,16]^ CRMO is a polymorphic disorder, therefore whole-body MRI is beneficial to demonstrate subclinical lesions.^[23]^ Bone biopsy remains the golden standard for diagnosis, especially in patients with a unifocal pattern for the differential diagnosis of malignancy.^[8]^ In the acute stage, osteoclastic bone resorption is predominantly carried out by neutrophils, while in the late stages, a mixed inflammatory response can be detected, including the presence of macrophages, lymphocytes, and plasma cells.^[14,23]^ The nonbacterial osteitis score can help guide the decision to perform a bone biopsy, with scores higher than 39 points indicating a high likelihood of CRMO diagnosis, while relatively lower possibility with score <28 points.^[3]^

Early identification of CRMO through noninvasive examinations and targeted diagnostic approaches can significantly reduce the suffering experienced by affected children. However, making an accurate diagnosis can be challenging due to the lack of specificity in clinical findings, laboratory tests, and radiology. Therefore, CRMO remains a diagnosis of exclusion. Two diagnostic criteria, the Jansson criteria and the Bristol criteria (Table 2), have been proposed.^[12,24]^ Several other conditions can present similar clinical and imaging features as CRMO, especially infectious osteomyelitis and Langerhans cell histiocytosis, while they displayed typical symptoms.^[25]^ For example, infectious osteomyelitis typically involves systemic symptoms, elevated ESR and CRP, and positive blood and bone cultures. Langerhans cell histiocytosis, on the other hand, often presents with systemic involvement, including bone, skin, and the central nervous system. Single lesions of bone involvement are frequently observed, primarily affecting the axial bones and skull.^[26]^ Abnormal Langerhans histological infiltration could be detected.

**Table 2:** Two diagnostic criteria.

Jansson criteria for diagnosis of CRMO:	
Major criteria	Minor criteria
1. Radiologically proven osteolytic/-sclerotic bone lesion.	A. Normal blood count and good general state of health.
2. Multifocal bone lesions.	B. CRP and ESR mildly-to-moderately elevated.
3. Palmoplantar pustulosis (PPP) or psoriasis.	C. Observation time > 6 months.
4. Sterile bone biopsy with signs of inflammation and/or fibrosis, sclerosis.	D. Hyperostosis.
	E. Associated with other autoimmune diseases apart from PPP or psoriasis.
	F. Grade I or II relatives with autoimmune or autoinflammatory diseases.
Threshold of diagnosis: ≥2 major criteria or 1 major plus 3 minor criteria.	

Bristol criteria for diagnosis of CRMO	
Required	Plus one of the following
1. The presence of typical clinical findings (bone pain+/-±localized swelling without significant local or systemic features of inflammation or infection).	A. >1 bone (or clavicle alone) without significantly raised CRP (<30 mg/L).
2. The presence of typical radiological findings (plain X-ray: showing a combination of lytic areas, sclerosis, and new bone formation, or preferably STIR MRI: showing bone marrow edema+/-±bone expansion, lytic areas, and periosteal reaction).	B. If unifocal diseases (other than clavicle) or CRP > 30 mg/L, with bone biopsy showing inflammatory changes (plasma cells, osteoclasts, fibrosis, or sclerosis) with no bacterial growth while not on antibiotic therapy.
Threshold of diagnosis: Both 1 and 2, plus A OR B.	

The treatment of CRMO is not standardized, with the dual objectives of pain management and preserving normal bone growth. NSAIDs are the first-line treatment option, but may lead to relapses in many cases.^[3,27]^ Second-line treatments include corticosteroids, TNF-α inhibitors, DMARDs, and bisphosphonates, etc.^[11,28]^ Bisphosphonates are powerful inhibitors of osteoclastic bone resorption and have been proven to be effective in CRMO treatment. They become necessary to induce remission and prevent bone damage in cases where NSAIDs alone fail to achieve clinical and radiological remission or when patients experience relapses after NSAID therapy, or cases with vertebrae involved.^[19]^ The patients we have reported underwent treatment with NSAIDs and biological agents, with case 3 specifically receiving additional bisphosphonate therapy. All patients exhibited favorable therapeutic outcomes, and subsequent follow-ups revealed no significant symptoms. Recently, the CNO/CRMO subgroup of the Childhood Arthritis and Rheumatology Research Alliance developed a consensus treatment plan with 3 treatment arms: (1) Conventional DMARDs such as methotrexate or sulfasalazine, (2) biologic DMARDs, including TNF-α inhibitors with or without concomitant methotrexate, and (3) bisphosphonates. Short courses of glucocorticoids are allowed in all regimens.

The overall prognosis of CRMO is generally favorable, although it tends to be prone to relapse, with an unpredictable clinical course.^[14,21]^ Early diagnosis and treatment are essential for improving outcomes. The pediatric chronic nonbacterial osteomyelitis score has been used to assess disease activity and improvement.^[29]^ In this report, 2 cases achieved pediatric chronic nonbacterial osteomyelitis scores of 70 or higher after 6 months of follow-up, and all 3 cases reached scores of 70 or higher after 12 months.

## 4. Conclusion

Diagnosing CRMO often poses challenges due to the absence of specific symptoms and laboratory markers, potentially leading to misdiagnosis or delayed diagnosis, which can subsequently result in recurrences, disease progression, or severe sequelae for affected children.^[8,30]^ This case report underscores the significance of 3 pediatric cases of CRMO, providing doctors with valuable hands-on experience in diagnosis and treatment while also serving as critical data for future investigations. By compiling and analyzing these cases, we can gain a deeper understanding of the underlying mechanisms, clinical manifestations, and therapeutic responses of CRMO. Ultimately, this knowledge will enable us to guide future clinical practices with greater precision and efficacy, ultimately reducing pain and suffering for children.

## Acknowledgments

The authors thank their colleagues at The Affiliated Hospital of Qingdao University for editorial support and comments.

## Author contributions

**Conceptualization:** Lin Liu, Ranran Zhang.

**Investigation:** Lin Liu.

**Writing** – **original draft:** Lin Liu.

**Writing** – **review & editing:** Lin Liu, Ranran Zhang, Yi Lin, Hong Chang.

**Data curation:** Nana Nie.

**Resources:** Nana Nie, Dahai Wang, Zhaisong Gao.
